# Operant conditioning of single units in rat motor cortex allows graded control of a prosthetic device

**DOI:** 10.1186/1471-2202-14-S1-O17

**Published:** 2013-07-08

**Authors:** Valerie Ego-Stengel, Pierre-Jean Arduin, Yves Frégnac, Daniel Shulz

**Affiliations:** 1 Unité de Neuroscience, Information et Complexité, UNIC-CNRS, Gif-sur-Yvette, 91190, France

## 

Operant control of a prosthesis by neuronal cortical activity is one of the successful strategies for implementing brain-machine interfaces, by which the subject learns to exert a volitional control of goal-directed movements.

Here, several motor cortex neurons were recorded simultaneously in head-fixed awake rats and were trained, one at a time, to modulate their firing in order to control the speed and direction of a 1D actuator carrying a water bottle. In the first phase of the experiment, the bottle could only move in one direction and this was triggered by an increase in firing rate. Most neurons submitted to this conditioning successfully increased their activity during trials, and this effect was enhanced across sessions. Once trained, the neuron chosen to control the operant behavior reacted consistently more rapidly than the other recorded neurons after trial onset. We observed also that the firing rate variability increased in an anticipatory way before trial onset, specifically for the neurons that could be conditioned successfully. However, this effect was observed only in the initial phases of the conditioning.

In the second phase of the experiment, neurons modulated their firing rate up or down in order to control the direction and speed of the water bottle. The bottle could thus move bilaterally, and the goal was to maintain the bottle in front of the rat's mouth in order to allow drinking. All conditioned neurons adapted their firing rate to the instantaneous bottle position so that the drinking time was increased relative to chance. The mean firing rate averaged over all trajectories depended on position, so that the mouth position operated as an attractor (at least for the bottle starting side). Again, the conditioned neuron reacted on average faster than the other neurons and led to a better bottle control than if trajectories were simulated using the activity of simultaneously recorded neurons.

Overall, our results demonstrate that conditioning single neurons is a suitable approach to control a prosthesis in real-time, and that these neurons occupy a lead position after learning, acting as "master" neurons in the network.

